# Identification of Fast-Evolving Genes in the Scleractinian Coral *Acropora* Using Comparative EST Analysis

**DOI:** 10.1371/journal.pone.0020140

**Published:** 2011-06-20

**Authors:** Akira Iguchi, Chuya Shinzato, Sylvain Forêt, David J. Miller

**Affiliations:** 1 Sesoko Station, Tropical Biosphere Research Center, University of the Ryukyus, Okinawa, Japan; 2 Okinawa Institute of Science and Technology, Okinawa, Japan; 3 ARC Centre of Excellence for Coral Reef Studies and Coral Genomics Group, School of Pharmacy and Molecular Sciences, James Cook University, Townsville, Australia; J. Craig Venter Institute, United States of America

## Abstract

To identify fast-evolving genes in reef-building corals, we performed direct comparative sequence analysis with expressed sequence tag (EST) datasets from two acroporid species: *Acropora palmata* from the Caribbean Sea and *A. millepora* from the Great Barrier Reef in Australia. Comparison of 589 independent sequences from 1,421 *A. palmata* contigs, with 10,247 *A. millepora* contigs resulted in the identification of 196 putative homologues. Most of the homologous pairs demonstrated high amino acid similarities (over 90%). Comparisons of putative homologues showing low amino acid similarities (under 90%) among the *Acropora* species to the near complete datasets from two other cnidarians (*Hydra magnipapillata* and *Nematostella vectensis*) implied that some were non-orthologous. Within 86 homologous pairs, 39 exhibited dN/dS ratios significantly less than 1, suggesting that these genes are under purifying selection associated with functional constraints. Eight independent genes showed dN/dS ratios exceeding 1, while three deviated significantly from 1, suggesting that these genes may play important roles in the adaptive evolution of *Acropora*. Our results also indicated that CEL-III lectin was under positive selection, consistent with a possible role in immunity or symbiont recognition. Further studies are needed to clarify the possible functions of the genes under positive selection to provide insight into the evolutionary process of corals.

## Introduction

One of the main aims of evolutionary biology is to understand the genetic basis of adaptive change [1], [2]. Genes associated with adaptive evolution often show relatively high rates of evolutionary change and are under selection [3], [4]. This is often inferred from the number of nonsynonymous substitutions per nonsynonymous site (amino acid replacement changes; dN) significantly exceeding the number of synonymous substitutions per synonymous site (silent changes; dS), an indication of accumulated amino acid changes (so-called “fast-evolving genes”). Although there is no a priori reason to expect genes showing higher levels of amino acid change to play important roles in morphological and developmental processes [5], positive selection has often been detected in genes involved in the co-evolutionary race, such as immune response, host-pathogen interactions, and reproduction – processes where protein-protein interactions are involved [6]–[8]. Therefore, the identification of fast-evolving genes can contribute to the understanding of the genetic bases of adaptive evolution, and thus provide insights into ecological niche partitioning and phenomena involved in the co-evolutionary race.

*Acropora* (Scleractinia, Cnidaria) is one of the most widespread, abundant, and species-rich of coral genera [113]–[180 species; 9,10]. *Acropora* species are highly diverse in terms of both morphological and ecological characteristics (e.g., habitat depth [10]). However, previous studies using neutral molecular markers were unable to detect fixed species-specific genetic differences among most *Acropora* species, likely due to introgressive hybridization or incomplete lineage sorting [11]–[13]. In sea urchins, the bindin protein is one of the dominants of gamete specificity and has been shown to be subject to positive selection [14]. Molecular markers based on the bindin gene delineate species boundaries more clearly than do other (neutral) molecular markers due to the rapid coalescence within a species [15]. In the same way, fast-evolving genes may provide some insight into the evolutionary process in the case of corals such as *Acropora*. In addition, the identification of molecules involved in symbiosis and pathogen recognition are likely to be fundamental importance in terms of understanding the genetic bases of coral bleaching and diseases which are the threats to the survival of corals [16]. Given that in the other animals the molecules involved in these processes are under positive selection, fast-evolving genes are good targets to uncover the molecular mechanisms of these phenomena in corals.

To date, there have been only three reports of genes under positive selection from corals - fluorescent proteins [17], ferritin [18] and tachylectin-2 [19]. However, the availability of significant bodies of expressed sequence tag (EST) data for two *Acropora* species (*A. millepora*, *A. palmata*; 18,20) provides an opportunity to perform more extensive searches for fast-evolving genes in this genus [16].

To this end, we have conducted the first extensive search for fast-evolving genes in the coral *Acropora* using direct comparative sequence analysis based on two *Acropora* EST datasets, focusing to some extent on taxonomically restricted genes because such genes might be involved in lineage- or species-specific traits [21] and thus provide species-specific molecular markers. In addition, we examined whether the CEL-III lectin is also under positive selection in *Acropora* on the basis that this gene may be involved in immunity or symbiosis in *A. millepora* [22], either of which might lead the gene to be under positive selection.

## Materials and Methods

### Selection of candidate sequences, BLAST and positive selection analyses

Using 1421 Cap3 assembled EST sequences based on 4,017 ESTs from *A. palmata* (http://compagen.zoologie.uni-kiel.de/datasets.html), BLASTX analysis was performed (e-value cut-off 1e^−5^) against the GenBank (NR: 976 hits) and Swissprot (manually curated protein sequences: 139 hits) databases. On the basis of these searches, a total of 149 *A. palmata* contigs matching only sequences of unknown or unnamed proteins (13) and hypothetical ones (136) were selected for further analysis. In addition, 440 *A. palmata* contigs with no significant matches in either database were selected, on the basis that these taxonomically restricted genes might be involved in coral-specific traits. The resulting set of 589 non-redundant *A. palmata* contigs were compared (using TBLASTX [23]) to the *A. millepora* (10,247 ESTs), *Nematostella vectensis* (166,595 ESTs), and *Hydra magnipapillata* (163,221 ESTs) EST datasets using the COMPAGEN comparative genomics platform (http://compagen.zoologie.uni-kiel.de/datasets.html). An e-value<10^−10^ was adopted as a cutoff for presumed orthology. Levels of amino acid sequence similarity were based on TBLASTX results.

Correct frame positions were estimated based on nucleotide sequences that were translated correctly in each of *A. palmata* and *A. millepora* through the following website: http://www.vivo.colostate.edu/molkit/translate/. dN and dS values of the nucleotide sequences of the open reading frames (ORFs) from both species were calculated using the sequences for which correct translations were possible for both species. To assess whether the dN/dS ratio was significantly different from 1, the Nei-Gojobori Jukes-Cantor method [24] was implemented, which uses a Z-test to determine whether dN/dS is significantly different from 1. These analyses were performed using MEGA ver. 4 [25]. Domain searches were performed using the Pfam database (http://pfam.sanger.ac.uk/search).

### CEL-III lectin analysis

Previously, two CEL-III lectins were reported from *A. millepora* [22]. Based on these sequences, we identified two CEL-III lectins in *A. palmata* (CL1132Contig, CL2457: http://compagen.zoologie.uni-kiel.de/) and one in *A. hyacinthus* (isotig11646: http://www.bio.utexas.edu/research/matz%5Flab/matzlab/Data.html). The five coral CEL-III lectin sequences were aligned with that of the sea cucumber CEL-III (accession number: AB109017) using ClustalW [26]; in some cases complete ORFs were not available, but the available data (139–144 amino acids) encompass most of the protein sequence. The Clustal alignment was visualised using the Boxshade server at: http://www.ch.embnet.org/cgi-bin/BOX_form_parser.

To perform phylogenetic analysis, the best-fit model of protein evolution was selected by ProtTest 2.4 [27], and the JTT+G model [28] was selected using the Akaike information criterion. Maximum likelihood (ML) analysis was performed by PhyML 3.0 [29]. The starting trees were computed using BioNJ and the topologies were optimized by nearest neighbour interchange and sub-tree pruning and regrafting. Support for each phylogenetic tree was tested by bootstraping (100 replicates). Bayesian analyses were performed using MrBayes v3.1.2 [30]. We conducted the analyses by 2 runs with 4 chains from a random starting tree that was run for 2,000,000 generations. The log-likelihood scores stabilized after 10,000 generations within and among these four independent analyses. Therefore, we discarded the initial 10,000 generations from each run and sampled 1 of every 100 generations from the remaining 1,990,000 generations (19,901 trees: across all four independent analyzes) to calculate posterior probabilities for each branch in the Bayesian tree. Based on the phylogenetic tree, with three *Acropora* CEL-III lectins (CL1132Contig, A049E7, and isotig11646) we performed site model analysis by CODEML, implemented in PAML v4.2 [31] using simple and complex models (M0: NSsites = 0; M1a: NSsites = 1; M2a: NSsites = 2; M7: NSsites = 7; M8: NSsites = 8; M8a: NSsites = 8, fix_omega = 1, omega = 1). Likelihood ratio tests (LRTs) were performed to compare two models. The negative of twice the difference between models (−2Δ(Log likelihood)) was used to approximate the χ^2^ value and the LRT was conducted by comparing −2Δ(Log likelihood) to the χ^2^ distribution with the degree of freedom estimated as the difference of parameters between models (critical values to be 5.99 and 9.21 at 5% and 1%; df = 2). When the LRT was significant, a Bayes Empirical Bayes (BEB) was used to identify amino acid sites under positive selection.

## Results

### Direct comparative sequence analysis of cnidarian ESTs

**Figure 1:**
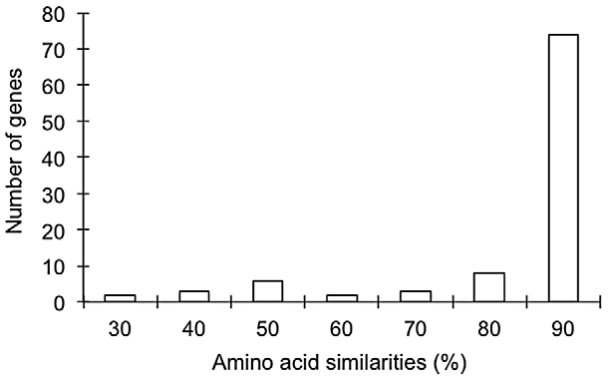
Distribution of the number of amino acid similarities in 98 *Acropora millepora*–*A. palmata* homologous pairs.

Of the 589 *A. palmata* contigs matching only unknown or unnamed proteins, hypothetical ones or with no significant non-cnidarian matches in the databases, 196 had putative orthologs in *A. millepora*. The amino acid similarities of 98 gene pairs for which correct translation positions were available (longer translation) were examined. The amino acid similarities among these homologous pairs ranged from 33.6% to 100% (average 89.5%; Fig. 1), with 74 and 24 sequences demonstrating greater than and less than 90% similarity, respectively (Fig. 1). Sequences giving low amino acid similarities between *Acropora* species were subjected to BLAST analysis performed against the EST datasets of *Hydra magnipapillata* and *Nematostella vectensis*. Several of the 13 *A. palmata* matching only unknown, unnamed, or hypothetical proteins and with <90% amino acid similarity with *A. millepora* were found to have better matches in *H. magnipapillata* and *N. vectensis* than in *A. millepora* and were hence excluded from further consideration (Table 1; <76.8% amino acid similarities in *A. millepora*).

**Table 1:** Amino acid similarity and E value of ESTs between *Acropora palmata* and *A. millepora*, *Hydra magnipapillata*, *Nematostella vectensis*.

Number of *A. palmata* sequence	Sequence length (amino acids)	*A. millepora*		*H. magnipapillata*		*N. vectensis*	
		Amino acid similarity (%)	E value	Amino acid similarity (%)	E value	Amino acid similarity (%)	E value
CL30Contig1	101	47.5	4E-27	**53.8**	2E-35	**65.3**	2E-41
CL428Contig1	75	50.7	1E-23	**52.0**	2E-22	**70.9**	1E-47
CL441Contig1	131	33.6	2E-19	**60.4**	8E-59	**68.4**	2E-67
CL505Contig1	35	51.4	1E-11	42.0	5E-11	**61.3**	3E-62
CL527Contig1	73	47.9	1E-19	**68.6**	1E-92	**56.9**	3E-78
CL663Contig1	74	44.6	2E-15	**49.6**	2E-49	**73.6**	6E-45
CL717Contig1	34	88.2	2E-12	N/A	N/A	37.1	7E-14
CL831Contig1	95	76.8	1E-108	32.6	7E-19	N/A	N/A
CL850Contig1	83	59.0	1E-29	**69.3**	3E-33	56.6	2E-24
CL957Contig1	90	88.9	1E-145	32.8	7E-12	40.0	3E-18
DR983392.1	100	39.0	2E-23	**51.1**	2E-44	**52.3**	8E-37
DR985484.1	35	51.4	1E-10	**55.6**	3E-43	**61.3**	2E-60
DR986161.1	99	83.8	1E-102	N/A	N/A	N/A	N/A

N/A means that putative EST could not be available due to an E value>10^−10^. Bold font indicates a higher amino acid similarity to *H. magnipapillata* or to *N. vectensis* than to *A. millepora*.

**Figure 2:**
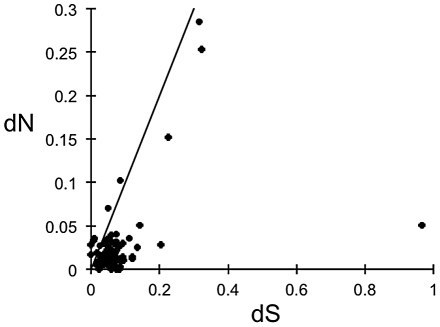
Number of nonsynonymous substitutions per nonsynonymous site (dN) plotted against the number of synonymous substitutions per synonymous site (dS) in 86 *Acropora millepora*–*A. palmata* putative homologous pairs. The line shows the neutral expectation (dN = dS).

To identify genes likely to be under positive selection, dN and dS values were calculated based on 86 putatively orthologous pairs of sequences from the two *Acropora* species (hypothetical protein: 52 exceeding 76.8% amino acid similarity; no-hit: 34 exceeding 72.7%). The distribution of dN and dS from the 86 homologous pairs is shown in Figure 2. Almost all of the homologous pairs (78) showed a dN/dS ratio <1. Among these, 39 homologous pairs had dN/dS ratios significantly <1. Eight independent homologous pairs had dN/dS ratios >1 and three (CL226Contig1, CL338Contig1, and CL504Contig1, all of which showed no-hit) exhibited a significant deviation from 1 (Table 2). The proteins encoded by these eight candidate genes contain no known domains on the basis of comparison with the Pfam database. The average of dN/dS values was 0.424. Twenty five homologous pairs had dN/dS ratios >0.424 and 6 ones of these (CL7Contig1, CL16Contig1, CL226Contig1, CL338Contig1, CL504Contig1) were significantly deviated from 0.424.

**Table 2:** Positive selection candidates of *Acropora*.

Number of *A. palmata* sequence	BLAST annotation	Amino acid similarity (%)	dS	dN	dN/dS
CL7Contig1	no-hit	72.7	0.049	0.07	1.4
CL226Contig1	no-hit	92.0	0.01	0.036	*3.6
CL338Contig1	no-hit	82.4	0.007	0.033	*4.7
CL504Contig1	no-hit	94.6	0	0.017	*N/A
CL717Contig1	hypothetical protein LOC570025 [Danio rerio]	88.2	0	0.028	N/A
CL727Contig1	hypothetical protein BRAFLDRAFT_99787 [Branchiostoma floridae]	97.8	0.016	0.019	1.2
CL831Contig1	hypothetical protein BRAFLDRAFT_264236 [Branchiostoma floridae]	76.8	0.085	0.102	1.2
DR986161.1	hypothetical protein MGL_2003 [Malassezia globosa CBS 7966]	83.8	0.026	0.027	1.0

Significance level: *0.05.

### The CEL-III lectin is under positive selection

**Figure 3:**
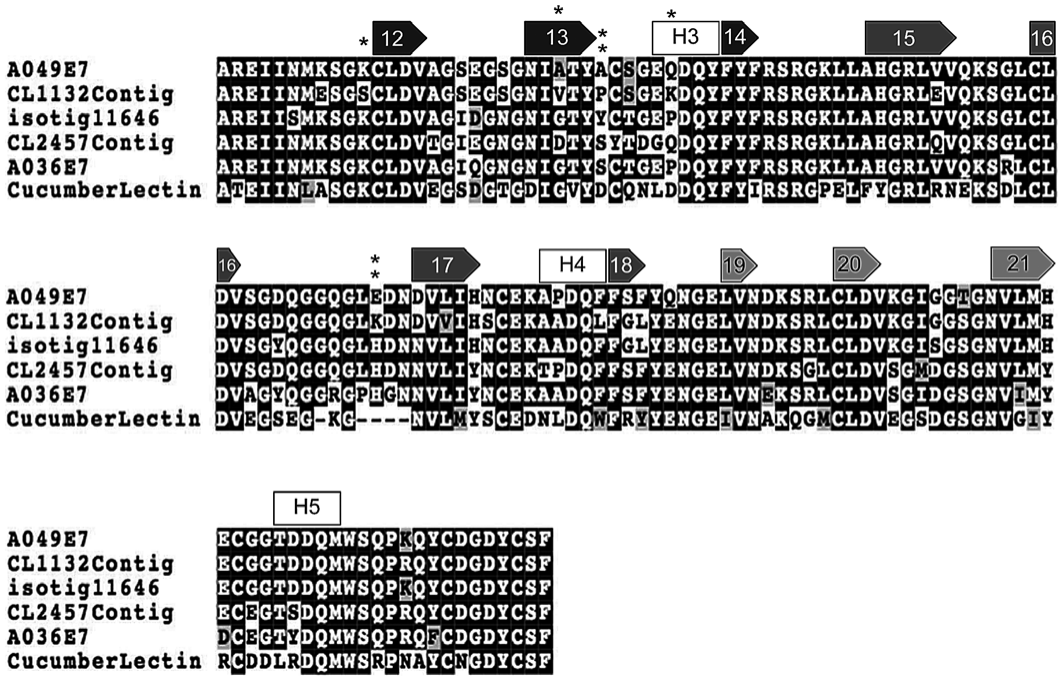
Boxshade alignment of five coral CEL-III lectins and sea cucumber CEL-III. Dark grey boxes are β-strands of subdomain 1γ, grey boxes are those of subdomain 2α, and bright grey boxes are those of subdomain 2γ. White boxes show 3_10_ helices. The numbers of boxes are in accordance with Uchida et al. [34].

**Figure 4:**
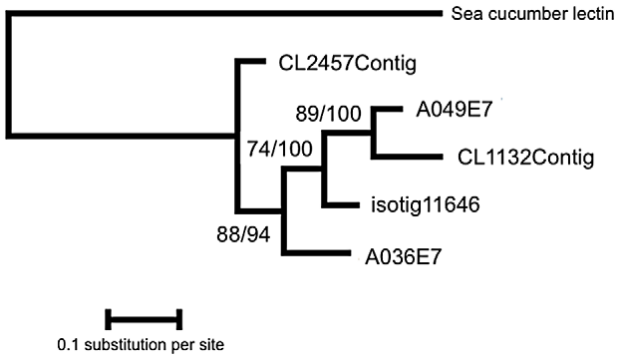
Phylogenetic relationship of CEL-III lectins inferred from ML and Bayesian analyses. Numbers beside nodes indicate ML/Bayesian posterior bootstrap probabilities.

Comparison of the CEL-III lectin dataset implies that A049E7 (*A. millepora*), CL1132Contig (*A. palmata*), and isotig11646 (*A. hyacinthus*) are orthologous (Figs. 3 and 4). Based on previous phylogenetic analyses of the genus *Acropora* species [12], the Caribbean species *A. palmata* forms an outgroup to Indo-Pacific *Acropora* species including *A. millepora* and *A. hyacinthus*. Thus, based on the alignment of the three putatively orthologous *Acropora* CEL-III lectins (CL1132Contig, A049E7, and isotig11646), site model analysis was performed, which implied that *Acropora* CEL-III lectins are under significant positive selection (Table 3). The models that allow ω>1 were statistically supported in the comparison of M1a and M2a (−2Δ(Log likelihood) = 14.494, df = 2, p<0.01) and M7 and M8 (−2Δ(Log likelihood) = 14.594, df = 2, p<0.01) at 99% confidence. BEB analysis identified five amino acid sites under positive selection (Table 3), one of which was located near the 12β-strand of subdomain 1γ (Fig. 3; [34]). Three sites under positive selection were concentrated around the 13β-strand and H3 3_10_ helix of subdomain 1, and another was near the 17β-strand of subdomain 2α.

**Table 3:** Log-likelihood values and the estimates of parameters under models with variable ω ratios in three coral CEL-III genes.

Model	Number of parameter	Estimates of parameters	Log likelihood	Sites under positive selection
M0	1	ω = 3.208	−754.733	-
M1a	2	p0 = 0.103 (p1 = 0.897)ω0 = 0.000, (ω1 = 1.000)	−757.011	-
M2a	4	p0 = 0.000, p1 = 0.883 (p2 = 0)ω0 = 0.000, (ω1 = 1.000), ω2 = 16.978	−749.764	**28A**, **72E**
M7	2	p0 = 1.39654, q = 0.005	−757.061	-
M8	4	p0 = 0.884, (p1 = 0.116)p = 2.012, q = 0.00500, ω = 16.978	−749.764	11K, 25A, **28A**, 33Q, **72E**
M8a	4	p0 = 0.103, (p1 = 0.897)p = 0.006, q = 1.113, ω = 1.000	−757.011	-

Bold characters show significant sites at 99% confidence. Other sites are significant at 95% confidence.

## Discussion

The direct comparative sequence analysis of *Acropora* ESTs revealed that most of the homologous pairs are highly similar at the amino acid level (Fig. 1), although several homologous pairs showing lower amino acid similarities were also found (Fig. 1). Of 196 putatively orthologous pairs of sequences in the two *Acropora* species, 98 were selected for further analysis on the basis of unambiguous start codons and long ORFs in both cases. To examine the possibility that some of the sequence pairs with <90% amino acid similarity between the two were not orthologous, the *Acropora* sequences were subjected to BLAST analyses against the EST datasets of *Hydra magnipapillata* and *Nematostella vectensis*. Several of the *A. palmata* sequences had better matches in *H. magnipapillata* and *N. vectensis* than in *A. millepora* (Table 1) leading to these being excluded from further consideration. The absence of *A. millepora* orthologs of these *A. palmata* genes is presumably due to the incomplete nature of the public EST dataset for *A. millepora*. Of the 86 homologous pairs for which the dN and dS were examined, 39 showed dN/dS ratios significantly less than 1, suggesting that these genes are under purifying selection associated with functional constraints. Eight independent genes showed dN/dS ratios exceeding 1 and three exhibited a significant deviation from 1. These fast-evolving genes are ideal targets for examining the genetic bases of adaptive evolution of *Acropora*. Further research is needed to clarify the functional roles of these candidate genes under positive selection.

Our analysis showed that coral CEL-III lectins are under positive selection. CEL-III lectins have been reported from the sea cucumber *Cucumaria echinata* [32], *A. millepora* [22], and the hydrozoan *Clytia hemisphaerica* [33]. A crystal structure is available for the sea cucumber CEL-III [34], enabling us to infer how the sites under positive selection are likely to affect the structure and function of the CEL-III lectin. From the alignment of sea cucumber and coral CEL-III lectins, we found that most of the sites under positive selection were concentrated around the Ca^2+^ binding region of domain 1 of sea cucumber CEL-III, which is thought to play an important role in the recognition of carbohydrate chains (Fig. 3; [34]). Thus, changes in this region may be adaptive for CEL-III to recognize various types of carbohydrate structures of non-self cells.

Lectins have frequently been implicated in innate immunity [35]. Grasso et al. [22] reported that the A049-E7 CEL-III lectin is expressed on the side of *A. millepora* larvae and primary polyps that is exposed to the environment, suggesting that it may be involved in the recognition of microorganisms for self-defence. It has often been reported that immunity related genes are under positive selection [6]; thus, our results support a possible role of CEL-III lectins of *Acropora* in immunity. In corals, several studies suggest the involvement of lectin in coral-algal symbiosis [36], [37]. Sea cucumber CEL-III lectin is a Ca^2+^-dependent and galactose-specific lectin [32]. Wood-Charlson et al. [37] suggested that the α-galactose residue is one of the carbohydrates constituting potential recognition ligands for lectin/glycan interactions in symbiosis of coral larvae. Therefore, the *Acropora* CEL-III lectins may also be involved in coral-algal symbiosis, which could induce positive selection of *Acropora* CEL-III lectins via a co-evolutionary race. Kvennefors et al. [38] reported the existence of mannose-binding lectin in *A. millepora* and its possible role in pathogen and symbiont recognition as an ancient innate immune system [39]. The functional roles of coral CEL-III lectins in both immunity and symbiosis processes should be pursued in future studies.

In conclusion, by performing direct comparative sequence analysis with cnidarian EST datasets, we identified several candidate genes under positive selection. Although the dN/dS ratios of most homologous pairs were <1, the possibility that these homologs are fast-evolving genes cannot be ruled out that these homologues are non-fast-evolving gene candidates, because Caribbean and Indo-Pacific *Acropora* species are genetically distinct and deeply branched [12]. Thus, the hallmarks of positive selection might be obscured by neutral nucleotide changes. Given that evaluating dN/dS using average values of dN and dS is highly conservative [40], a more sensitive approach (e.g., site model analysis) with sequences of multiple species would be useful to assess whether genes showing higher dN/dS ratios are under positive selection or relaxed negative selection. There is also the possibility that the genes showing higher dN/dS may be just outliers that have become fixed due to small effective population sizes [5]. In fact, the effective population sizes of some *Acropora* species may be limited by high variation of reproductive success, periodic mass mortality by coral bleaching or predation by crown-of-thorns starfish [41], therefore, some coral species may have experienced demographic bottlenecks. The comparison of candidate genes under positive selection obtained using multiple neighbouring species are important both to clarify their significance in adaptive evolution of this genus as well as to assess whether these candidate genes are indeed orthologous between the two *Acropora* species examined in this study. The ongoing coral genome project (Shinzato, personal communication) will provide the opportunity to confirm that the target genes originate from coral itself rather than from other components of the association, and will allow the use of a more rigorous genome wide approach to surveying candidate genes under positive selection (Shinzato, personal communication). An important next step will be to investigate the expression patterns of these fast-evolving genes to better characterise their function and regulation.
